# Revisiting the Relationship Between Internal Focus and Balance Control in Young and Older Adults

**DOI:** 10.3389/fneur.2018.01131

**Published:** 2019-01-09

**Authors:** Victoria W. K. Chow, Toby J. Ellmers, William R. Young, Toby C. T. Mak, Thomson W. L. Wong

**Affiliations:** ^1^Li Ka Shing Faculty of Medicine, School of Public Health, The University of Hong Kong, Hong Kong, China; ^2^Department of Clinical Sciences, Brunel University London, London, United Kingdom; ^3^Institute for Environment, Health and Societies, Brunel University London, London, United Kingdom

**Keywords:** attention, internal focus, EEG, T3-Fz coherence, balance, aging

## Abstract

Research highlights the detrimental effect that directing too much conscious attention toward movement can have on postural control. While this concept has received support from many studies, recent evidence demonstrates that this principle does not always translate to aging clinical populations. Given the increasing clinical interest in this topic, the current study evaluated if the original notion (that an internal focus results in compromised balance performance) is upheld in young and older adults during a challenging balance task where we are able to objectively corroborate changes in attentional focus; using an electroencephalography (EEG) method previously identified as an objective indicator of conscious movement control. This method assesses the neural coherence, or “communication,” between T3 (verbal-analytical) and Fz (motor-planning) regions of the brain. Thirty-nine young and 40 older adults performed a challenging balance task while holding a 2-meter pole under two randomized conditions: Baseline and Internal focus of attention (directing attention internally toward movement production). Results showed that young adults demonstrated increased EEG T3-Fz coherence in conjunction with increased sway path during the Internal focus condition. However, no significant differences were observed in older adults between conditions for any measure. The current study provides supporting evidence for the detrimental effect that adopting an Internal focus can have on postural control—especially in populations able to govern these processes in a relatively “automatic” manner (e.g., young adults). However, this work illustrates that such observations may not readily translate between populations and are not robust to age-related changes. Further work is necessary to examine mechanisms underlying this clear translational issue.

## Introduction

Traditional conceptualizations have viewed postural control as a largely automatic process requiring minimal conscious involvement. However, recent decades have seen this notion become increasingly discredited, largely based on observations made in dual-tasking paradigms, where poorer performance on a range of different balance tasks are observed when carrying out a simultaneous cognitive task [for reviews see (1, 2)]. While dual-task related breakdown in postural control is often reported in young adults [e.g., (3, 4)], these decrements appear to be particularly pronounced in older adults (1). This observation has led researchers to propose the existence of an age-related increase in the minimum amount of controlled (conscious) processing required to effectively regulate postural stability (1). Therefore, while older adults *can* maintain similar levels of postural stability (compared to young adults) during conditions of single-task, doing so will likely require increased attentional resources necessary for such controlled processing—resources less available during dual-task conditions.

These findings illustrate that postural control, particularly in older adults, requires some level of conscious, attentional input. However, other lines of research highlight the detrimental effect that directing *too* much conscious attention toward movement can have on postural control. For example, seminal work published by Wulf and colleagues describes enhanced postural stability when performers direct attention externally (e.g., toward ensuring markers placed on a stabilometer remain horizontal), rather than internally (e.g., toward minimizing movement in the ankles) (5, 6). These findings have since been replicated during other experimental conditions designed to similarly limit the amount of conscious attention directed toward postural control in both young and older adults (7–9). This has led researchers to suggest that stability may be enhanced during balance tasks by promoting the use of more automatic control mechanisms (6, 7, 9). It would, therefore, appear that while the control of posture and gait does require some level of cognitive input (1, 2), these processes may typically be governed using largely automatic processes. As such, it has been suggested that adopting an internal focus of attention may disrupt the “automatic” processes typically used to regulate posture, leading to superfluous muscle activity and constrained motor outputs (10).

This perspective has received support from research carried out in various contexts, especially skilled sports performance [e.g., (10–12)]. However, recent evidence demonstrates that this principle does not always translate between contexts as expected and that the behavioral (e.g., performance) consequences of adopting an internal focus may depend on several factors, including the performer's level of skill/movement proficiency (13–16). For example, Castaneda and Gray (14) found that highly skilled baseball players benefited from an external focus during a batting simulation, whereas novices performed best when adopting an internal focus. In the context of rehabilitation after Stroke, compared to an external focus condition, adopting an internal focus has also been shown to enhance movement proficiency (17, 18), thus seemingly contradicting basic assumptions established through observations in young healthy adults completing identical tasks [e.g., (19)]. Kal et al. (18) argued that, similar to when novice performers attempt to learn a sporting skill for the first time, in situations where automatic processes lack the requisite “knowledge” to proficiently carry out the given task, it may be necessary to allocate attention toward the conscious control of a skill in order to avoid gross performance errors. In other words, in the absence of adequate automatic control processes, motor tasks that were once completed with relative ease now present a formidable challenge and command significant cognitive resources during their execution. As such, one might argue that changes in task difficulty are sufficient to determine whether the adoption of an internal focus represents an adaptive or maladaptive strategy.

When attempting to translate the above-described notions proposed by Wulf and colleagues (6, 10) to different clinical groups, this issue of task difficulty/increased reliance on conscious control processes is rarely noted and could explain discrepancies in findings observed between young healthy adults and clinical populations [e.g., (18, 19)]. Furthermore, in the absence of a validated method for objectively measuring the degree to which a performer is focussing internally, previous research on this topic has almost exclusively relied solely on experimental manipulations of, and/or self-reported changes in, attentional focus to rationalize observed changes in performance. Utilizing an objective, real-time measure would allow for further investigation into the mechanisms through which these aforementioned discrepancies may occur. For example, if certain clinical populations do, in fact, benefit from adopting an internal focus, then we would expect objective measures to record heightened levels of conscious movement processing at baseline.

Considering the above, and given the ever-increasing interest (from both researchers and clinicians) in this topic, the current study aimed to address two fundamental issues. First, we evaluated if the original notion proposed by Wulf and colleagues (5, 6, 10) is upheld in young adults during a challenging balance task where we are able to objectively corroborate changes in attentional focus using an electroencephalography (EEG) method capable of objectively measuring changes in conscious control processes (described below). Second, given discrepancies in observations made between young healthy adults and older adults with neurological conditions [e.g., (18, 19)], we aimed to evaluate if our observations in young adults are robust simply to age-related changes (i.e., without the added complexity of co-morbidities and neurological complexities). This was achieved by inviting an active and healthy cohort of older adults to complete the same protocol as the young adults, while attempting to normalize the difficulty of the balance task to account for age-related changes in balance control. Based on this task-difficulty normalization process, we predicted to observe similar patterns of behavioral outcomes in both the young and older adults—whereby the adoption of an internal focus results in disrupted regulation of postural stability. Furthermore, as this previous research tends to contrast conditions of internal focus with an external focus of attention, rather than exploring how directing attention internally alters postural control when compared to a baseline no-instruction condition [e.g., (5–8)], it is difficult to isolate the effects of adopting an internal focus. As such, the present research compared the effects of adopting an internal focus of attention to a baseline no-instruction condition.

Research suggests that EEG coherence, or “communication,” between T3 (verbal-analytical) and Fz (motor-planning) regions of the brain may provide an objective, real-time measure of attentional focus during postural control tasks. For example, Ellmers et al. (20) reported significantly higher T3-Fz coherence during a postural sway task when young adults consciously controlled their swaying movements (“internal focus” condition), compared to when attention was directed toward either an external auditory cue (“external focus” condition) or a baseline (no instruction) condition. Similarly, T3-Fz coherence when regulating postural stability has also been shown to increase in line with task difficulty (21). These findings suggest that consciously processing postural control may be characterized by increased conscious verbal-analytical or cognitive processes, thus supporting previous research which implicates verbal processes in the conscious control of posture and gait (22). These findings are also in line with the predictions presented in the Theory of Reinvestment [for a review, see (23)], which posits that conscious motor processing is characterized by an increased reliance on explicit verbal cues/rules. As such, while the regulation of postural stability typically occurs with low levels of explicit verbal-analytical processes, attempts to consciously control or monitor posture results in an increased reliance on such explicit processes. These results describing increased T3-Fz coherence during conditions of heightened conscious postural control also support those presented previously during other motor tasks (24, 25). For example, Zhu et al. (25) observed greater T3-Fz coherence during a golf putting task performed by individuals reporting a greater propensity for consciously controlling their movements. In a second experiment, the authors similarly reported increased T3-Fz coherence in individuals performing a golf putt under conditions designed to promote heightened conscious, cognitive involvement. Taken together, these findings suggest that conscious, controlled motor processing (including during postural control tasks) can be characterized by heightened EEG T3-Fz coherence—indicating increased verbal-analytical involvement during motor planning and control.

In the present study, healthy young and older adults completed a challenging postural control task under conditions of Baseline and Internal focus, while measuring changes in EEG T3-Fz coherence. We predicted to observe the following results in young adults: (1) Increased EEG T3-Fz coherence during Internal focus; (2) Decreased postural stability (i.e., increased sway) during Internal focus; (3) Significant positive associations between the change in T3-Fz coherence and postural sway between Baseline and Internal focus; (4) Significant positive associations between a trait propensity to consciously control and monitor movement, and both T3-Fz coherence and body sway during Baseline. Furthermore, as attempts were made to normalize the difficulty of the postural task for older adult participants, we predicted to observe the same patterns of results in a group of healthy older adults with high levels of functional balance, deemed to be at a low risk of falling.

## Methods

### Participants

Thirty-nine young adults (20 men and 19 women) aged between 18 and 39 years of age (*M* = 23.5 years, *SD* = 5.2 years), and forty older adults (12 men and 28 women) aged between 65 and 83 years (*M* = 69.7 years, *SD* = 3.8 years), participated in the research. Young adults were recruited from undergraduate and post-graduate courses in London and Hong Kong, while older adults were recruited from different elderly community centers in Hong Kong. The inclusion criteria for young adults were: (i) aged 18 or above and below 40 years; (ii) ability to provide written informed consent; (iii) ability to stand independently without any walking aid, and; (iv) no history of cerebral vascular disease, Parkinson's or any other neurological impairments. Inclusion criteria were identical for older adults, but with the following amendments/additions: (i) participants were aged 65 years or above; (ii) a score of 24/30 or above in the Chinese version of Mini-Mental State Examination [MMSE-C (26, 27)], and; (iii) a score of 45/56 or higher on the Berg Balance Scale [BBS (28)]. The older adult participants had a mean MMSE-C score of 29.23 (*SD* = 0.92) out of 30, and a mean BBS score of 54.88 (*SD* = 1.52) out of 56. These variables were not assessed in the young adult participants, given that young adults typically score 100% on both assessments. Two (out of 40) older adults reported that they did not engage in weekly exercise (compared to 5/39 young adults), and all but one older adult reported their health status as fair–excellent (compared to 38/39 young adults reporting their health status as fair–excellent).

Participants had no prior experience with the specific tasks utilized in the current protocol. The study protocol was approved by the Institutional Review Board of the University of Hong Kong/Hospital Authority Hong Kong West Cluster (HKU/HA HKW IRB).

### Task and Procedure

For young adults, the balance task required participants to stand as still as possible in tandem stance on a 19.7″ × 16.1″ × 2.4″ foam-pad (Balance Pad Elite, AIREX, Switzerland), while horizontally holding a 2-m pole. Participants held the pole with their hands facing upwards and elbows tucked against their body. Older adults undertook an identical procedure, the only difference being that they performed the balance task while standing with a narrow base of support (standing with their feet together), rather than in tandem stance. This methodological alteration was deemed necessary to ensure a comparable level of task difficulty between young and older adult participants, as pilot testing revealed that many older adult participants were unable to complete a 20s trial standing in tandem stance. As narrow-based standing is ranked as the next most challenging standing position, after tandem standing [according to the BBS (28)], this was deemed the most appropriate modified balance task for older adults to complete.

All participants performed two 20s trials under conditions of: Baseline (no instructions, other than to “stand as still as possible”), and; Internal focus (instructed to focus explicitly on lower limb movement: “Try to focus on your lower limb movement while performing the task”). Trials were presented in a randomized order. Participants fixated a point on a featureless wall 2-meters in front of them, with approximate head-pitch and general gaze fixation monitored and noted by the experimenter during each trial to ensure consistency between conditions^1^. The balance task and attentional focus instructions were derived from those previously used by Wulf et al. (6).

### Apparatus

Electroencephalographic activity was recorded using a wireless EEG device (Brainquiry PET 4.0, Brainquiry, The Netherlands) at a sample rate of 200 Hz. EEG data were recorded using real-time biophysical data acquisition software (BioExplorer 1.5, CyberEvolution, US). EEG activity was recorded from two scalp locations [T3 [verbal-analytical] and Fz [motor planning]; see Ellmers et al. (20)] referenced to the right mastoid and grounded to the left mastoid using disposable electrodes (ARBO H124SG Ø 24 mm, Kendall, US), in accordance with the standard international 10–20 system (29). An impedance test was conducted to ensure a sufficient signal-to-noise ratio before each measurement. EEG signals were pre-processed (low-pass filter: 42 Hz, high-pass filter: 2 Hz) to remove potential biologic artifacts. T3-Fz coherence was calculated in 1 Hz frequency bins throughout each trial, using algorithms previously described by Zhu et al. (25). Previous research (20) has demonstrated that alpha2 (10–12 Hz) T3-Fz coherence is sensitive at detecting within-subject changes in conscious movement processing/attentional focus during a postural sway task (while no such changes were observed for alpha1(8–10 Hz) T3-Fz coherence). Similar results were also presented by Chu and Wong (21), whereby only alpha2 T3-Fz coherence was sensitive at detecting increases in task difficulty during a postural stability task. These findings support those presented by Zhu et al. (25) which highlight T3-Fz coherence as being sensitive to detecting differences in conscious movement processing during a golf-putting task. Consequently, the main EEG variable of interest was alpha2 T3-Fz coherence, with alpha2 T3-Fz coherence averages calculated for each trial, and then averaged across the relevant conditions. EEG pre-processing and coherence calculations were conducted using custom scripts in a biophysical data processing and analysis software (BioReviewer 1.6, CyberEvolution, US).

Body sway data were collected using 3-D motion capture with a minimum capture frequency of 100 Hz, using reflective markers placed on participants' sternum. Postural sway was obtained by calculating the root-mean-square of sternum co-ordinates in the horizontal (X–Z) plane throughout the 20-s trial. Raw data were passed through a low-pass Butterworth filter with a cut-off frequency of 5 Hz and analyzed using custom Matlab (R2015B Mathworks Inc., Natick, USA) scripts to calculate the variable of total body sway (mm).

### Questionnaires

Participants' trait propensity to consciously process their movement was assessed using the Movement Specific Reinvestment Scale [MSRS; Masters et al. (30)]. This 10-item questionnaire consists of two 5-item subscales: conscious motor processing (“movement control”; e.g., “*I am always trying to think about my movements when I carry them out”*) and movement self-consciousness (“movement monitoring”; e.g., “*I'm self-conscious about the way I look when I am moving”*). Items are rated on a 6-point Likert scale (1 = *strongly disagree*; 6 = *strongly agree*). Both subscales range from 5–30, with higher scores reflecting a higher propensity for reinvestment. Both subscales have good internal validity and test-retest reliability (30).

### Data Analysis

#### Baseline-Internal Focus Changes

As the majority of the variables were non-normally distributed, it was not possible to run a 2 × 2 (Young/Older adults × Condition) ANOVA. Furthermore, while attempts were made to normalize the difficulty of the balance task between groups, we cannot ensure parity in the task difficulty. Therefore, we treated the young and older adult data as two separate datasets and analyzed them as such. For young adults, a paired-samples *t*-test was used to explore any differences in T3-Fz coherence between Baseline and Internal focus. For older adults, between-condition changes in T3-Fz coherence were examined using a Wilcoxin test. The use of a non-parametric test was deemed necessary here, and elsewhere, as data were non-normally distributed. Separate Wilcoxon tests were used to determine the Baseline-Internal focus change in total body sway for both young and older adults. For all statistical comparisons, effect sizes are reported as Cohen's *d*, unless the assumption of normality is violated, where effect sizes are reported as *r* = *Z*/√*N* (31).

#### Correlations

Separate bivariate correlations were used to investigate the association between the Baseline-Internal focus percentage change in both T3-Fz coherence and total body sway, in young and older adults. Separate bivariate correlations were also used to explore any associations between MSRS scores and either Baseline T3-Fz coherence or Baseline total body sway, in both young and older adults. All analyses were conducted with Spearman's non-parametric correlations (given the failures to meet parametric assumptions), aside from the correlation exploring MSRS scores and Baseline T3-Fz coherence in young adults.

## Results

### Young Adults

**Figure 1:**
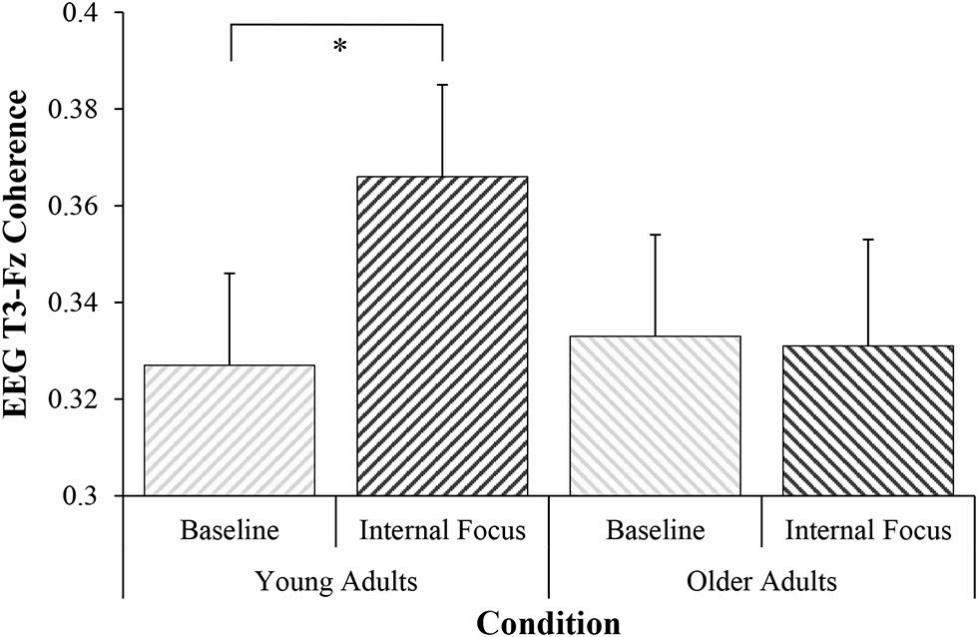
Electroencephalographic alpha2 (10–12 Hz) T3-Fz coherence during conditions of Baseline and Internal focus (mean ± standard error), ^*^p < 0.05.

**Figure 2:**
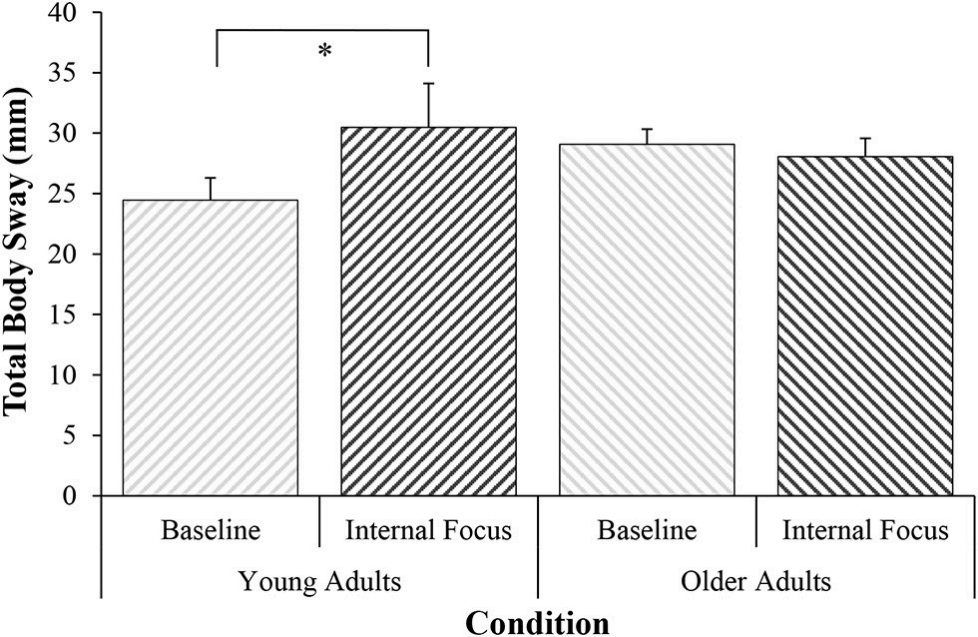
Total body sway (mm) during conditions of Baseline and Internal focus (mean ± standard error), ^*^p < 0.05.

**Figure 3:**
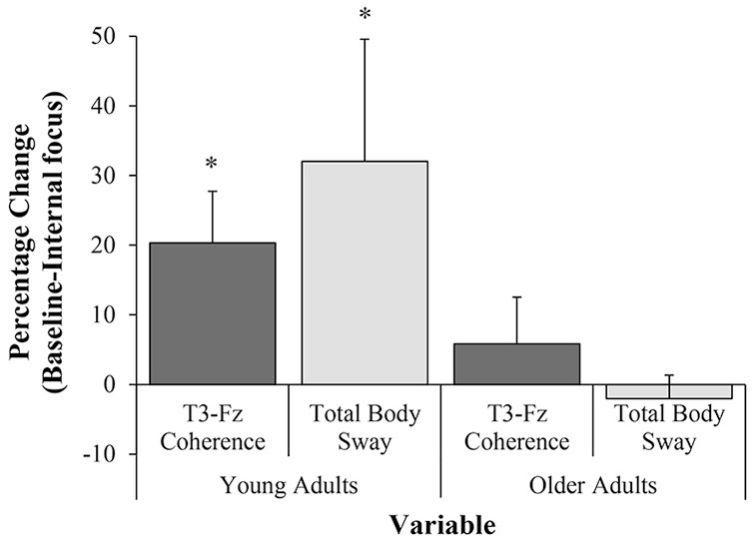
Mean percentage change (mean change ± standard error of the mean change) between Baseline and Internal focus in T3-Fz coherence and total body sway for young and older adults, ^*^percentage change significant to *p* < 0.05. Note: Positive values indicate an increase during Internal focus, compared to Baseline.

There was a significant increase in T3-Fz coherence from Baseline (*M* = 0.327, *SD* = 0.12) to Internal (*M* = 0.366, *SD* = 0.12), *t*_(38)_ = −2.07, *p* = 0.023, *d* = 0.33 (see Figure 1). Increased coherence was accompanied by significantly greater total body sway during Internal (*M* = 30.48 mm, *SD* = 22.68), compared to Baseline (*M* = 24.46 mm, *SD* = 11.50), *Z* = −1.76, *p* = 0.040, *r* = 0.28 (see Figure 2). Percentage change data for both analyses are presented in Figure 3.

### Older Adults

There was no significant change in T3-Fz coherence observed from Baseline (*M* = 0.333, *SD* = 0.13) to Internal (*M* = 0.331, *SD* = 0.14), *Z* = −0.36, *p* = 0.36, *r* = 0.06 (see Figure 1). There was a similar lack of significant change in total body sway observed between Baseline (*M* = 29.08 mm, *SD* = 7.98) and Internal (*M* = 28.06 mm, *SD* = 9.58), *Z* = −0.83, *p* = 0.20, *r* = 0.13 (see Figure 2). Percentage change data for both analyses are presented in Figure 3.

### Correlations

#### Percentage Change

There were no significant correlations observed between the percentage change in T3-Fz coherence and total body sway from Baseline to Internal in either young (*r* = 0.09, *p* = 0.30) or older adults (*r* = 0.13, *p* = 0.22).

#### Trait MSRS

In young adults, MSRS scores were not significantly correlated with either Baseline T3-Fz coherence (*r* = 0.11, *p* = 0.26) or Baseline total body sway (*r* = −0.05, *p* = 0.38). There were a similar lack of significant correlations observed in older adults between MSRS scores either Baseline T3-Fz coherence (*r* = −0.09, *p* = 0.30) or Baseline total body sway (*r* = −0.25, *p* = 0.06).

## Discussion

The current study provides strong support for the notion that adopting an internal focus of attention can disrupt performance of motor tasks that are typically governed by largely automatic processes—with young adults demonstrating greater postural sway during conditions of Internal focus. The current findings support the original notion proposed by Wulf and colleagues (5, 6, 10), in addition to numerous subsequent suggestions [e.g., (11, 12, 19)], that the adoption of an internal focus disrupts the ability to control motor performance in a task typically governed using largely automatic processes. While previous research has used EEG to infer alterations in attentional focus during a postural task (32), to our knowledge, this is the first instance where such associations have been demonstrated and compared between young and older adult groups.

We aimed to also evaluate whether this principle could be readily translated to clinical contexts by replicating the protocol in a cohort of older adults, while normalizing for task-difficulty. We had expected to observe comparable results in both our young and older adult cohorts, given both the older adults' relatively high-levels of physical functioning and the attempts to normalize task difficulty between-groups. However, any statistically significant effect of the internal focus manipulation (on both EEG T3-Fz coherence and postural sway) was restricted to young adults, as no significant changes in either measure were observed in older adults (see Figure 3).

### Observations in Young Adults

Given the weight of evidence supporting an association between internal focus of attention and disrupted motor performance on tasks normally regulated through “automatic” processes, any contradictory results would have been highly unexpected. Nevertheless, given the scale of recent and ongoing efforts to apply this perspective to various clinical (i.e., complex) contexts [e.g., (17, 18, 33)], it was important to re-establish these fundamental associations using an objective corroboration of the attention manipulation used. We suggest that our current observations in young adults fulfill this objective and, while further research is necessary to better-establish underlying mechanisms mediating this relationship, our findings help to establish a foundation from which we can evaluate the degree to which such associations translate to other contexts and populations.

It is important to note that, when calculating the percentage change in EEG T3-Fz coherence and postural sway between Baseline and Internal conditions, no significant correlation was found between these two variables. In light of previous validations of the EEG protocol (20, 21, 25) and the clear changes observed in EEG T3-Fz coherence between conditions in young adults indicating a greater reliance on cognitive verbal-analytical processes to regulate motor output, we suggest that the lack of any statistical association between metrics indicating percentage change cannot be primarily due to poor sensitivity in the EEG T3-Fz measurement. As such, despite our results highlighting an increase in both conscious movement processing and disrupted postural stability during conditions of internal focus in young adults (see Figure 3), the current data show no evidence for the concept that the degree of increased conscious control is associated with magnitude of behavioral change.

According to the basic principle that a propensity to consciously control movement will jeopardize movement automaticity and compromise motor performance, one would also expect to observe an association between MSRS scores—a trait measurement of an individual's propensity to consciously control and monitor their movement—and total body sway at Baseline. Our results, however, show no such association. In contrast to the clear support the current results (in young adults) show for the seminal findings of Wulf and colleagues (5, 6), the lack of any significant correlation observed concerning the MSRS raises important concerns about whether simple measures of dispositional traits can be expected to associate with complex attentional processes across a range of tasks. This proposal is further supported by a similar lack of association between MSRS scores and EEG T3-Fz coherence during Baseline—results in line with previous research which demonstrates a lack of between-group difference (based on MSRS scores) in EEG T3-Fz coherence (20, 21). While previous research has suggested a weak positive association between the MSRS and postural sway in young adults during a simple, quiet standing task whereby participants were instructed to stand as still as possible while standing in a comfortable, self-selected stance (34), no such associations were evident in older adults. This led the authors to suggest that scores on the trait measure of the MSRS may not necessary reflect the true amount of conscious involvement that individuals will “reinvest” into postural control—and instead propose that “…state measures of conscious movement processing (i.e., using MSRS as a context specific measure or assessing neural activity)” [(34), p. 448] may provide a more accurate indication of state processes.

### Observations in Older Adults

We predicted that significant Baseline-Internal focus increases in both EEG T3-Fz and postural sway observed in younger adults (see Figure 3) would also be observed in older adult participants. Considering that attempts were made to normalize task difficulty between groups and the circumstance that the cohort of older adults were relatively highly functioning both in cognition and physical status, we saw no clear reason to expect findings to contradict those observed in young adults. The lack of significant results observed in our older adult cohort is therefore surprising, as previous research suggests that adopting an internal focus of attention may disrupt the “automatic” processes typically used to regulate posture, leading to superfluous muscle activity and constrained, less effective motor outputs in both young and older adults (10). However, it is worth noting that this previous research tends to contrast conditions of internal focus with an external focus of attention, rather than exploring how directing attention internally alters postural control when compared to a baseline no-instruction condition [e.g., (5–8)]. As such, it is possible that these previous results are a consequence of the positive impact of an external, rather than a negative effect of an internal focus of attention—an idea supported by findings presented by Richer et al. (9). The internal focus manipulation did, however, negatively impact young adults' regulation of postural stability. The lack of significant effect on either EEG T3-Fz coherence or postural sway was only observed in the older adult sample. We offer several speculations below in an attempt to rationalize these null results.

It is possible that our cohort of older adults adopted an internal focus of attention during Baseline trials, thus reducing the potential for change between conditions. For example, while the young adults may have been able to achieve the task of “standing as still as possible” with relatively “automatic” postural control processes, it is possible that such instructions may have induced a more conscious strategy of postural control in the older adult sample. This would support the notions presented previously by Boisgontier et al. (1), who suggest an age-related increase in the level of controlled conscious processing needed to regulate postural stability. However, we suggest this to be unlikely, due to the identical between-group values in Baseline EEG T3-Fz coherence (*young adult M* = 0.33, *SD* = 0.12; *older adult M* = 0.33, *SD* = 0.13). It is, however, possible that while EEG T3-Fz coherence is sensitive at detecting within-subject change in conscious movement processing during postural tasks [as indicated by the significant increase in coherence observed between Baseline and Internal focus in young adults in the present study, in addition to results presented previously by both Chu and Wong (21) and Ellmers et al. (20)], this method lacks sensitivity for detecting between-subject differences in internal focus. This could, potentially, account for the lack of association between MSRS scores and EEG T3-Fz coherence observed both in the present research and in previous studies (20, 21).

Another suggestion for the lack of comparable (to young adults) Baseline-Internal focus change in older adults relates to the potential between-group differences in how these instructions were interpreted and subsequently utilized to regulate posture. For example, Mak and colleagues (35, 36) have found evidence to suggest that the manner with which older adults alter their behavior following the adoption of an internal focus was dependent on previous experiences with falling—with these experiences resulting in different interpretations of the internal focus instructions. For example, fallers might instinctively think about significant and problematic factors that jeopardize their balance on a daily basis, whereas their non-falling counterparts may be more inclined to focus attention on more generic movement rules. In other words, the manifestation of internal focus will likely be highly personalized and dependent on the unique interaction of traits and experiences present within each individual. While such individual differences will inevitably be present in young adults, we speculate that such differences are likely to be compounded by increased age and associated decline in automatic postural control mechanisms (1). Consequently, it is possible that while young adults relied on generic, explicit movement rules to control posture during Internal focus, their older adult counterparts instead adopted a more individualistic approach—which, for example, may have included ruminations unrelated to the conscious, cognitive control of posture and, thus, unlikely to have been registered through T3-Fz coherence. Regardless of the specific reasons, we must conclude that the basic notions proposed by Wulf and colleagues (5, 6, 10) cannot be assumed to readily translate to clinical contexts, even within a relatively simple “static” balance task.

In the complex attentional processes that have, hitherto, frequently been categorized as representing either an “external” or “internal” focus, the scope for between-subject differences are vast, especially when considering complexities associated with increased age and/or neurological impairment. To evaluate these complex processes we need to isolate and categorize the various multifaceted cognitive and attentional processes pertinent to different cohorts/conditions and associate changes in these measures with behavioral metrics indicative of both movement efficiency and efficacy. We anticipate that failure to acknowledge and evaluate these complex mechanisms will lead to the continued emergence of conflicting results, as identified by Kal et al. (18).

## Limitations

This study is not without its limitations. Firstly, the study failed to measure self-reported changes in attentional focus. As such, it is possible that the lack of behavioral change observed in older adult participants was simply due to these participants simply failing to successfully engage in the manipulation and direct attention internally. We suggest that this is unlikely, as these internal focus instructions were derived from, and identical to, previous research demonstrating significant behavioral effects [e.g., (5, 6, 10)]. Another potential limitation of this research relates to the possibility that there were between-condition differences in the level of attention allocated toward the postural task. For example, as the task involved participants standing in a challenging stance whilst holding a 2-m pole, it is possible that the differences observed between how young and older adults responded to the internal instructions may have been a consequence of differences in Baseline levels of task prioritization: Whilst the older adults may have been focused entirely on maintaining postural stability, it is possible that the young adults were also directing attention toward minimizing the movement of the pole. However, given that the postural task was designed to be challenging, and participants were instructed to “stand as still as possible,” we deem it unlikely that participants would have been directing explicit attention toward the pole at the expense of maintaining postural stability.

## Conclusions

The current study provides further support for the concept that adopting an internal focus of attention disrupts motor performance in tasks typically considered to be largely automatic. To our knowledge this is the first instance where such associations have been demonstrated in conjunction with an objective corroboration of the Internal focus condition; in this instance this was represented as an increase in EEG T3-Fz coherence. We aimed to evaluate whether this principle could be readily translated to clinical contexts by replicating the protocol in a cohort of older adults, while normalizing for task-difficulty. When instructing older adults to adopt an internal focus of attention during the balance task, we observed a lack of significant change in both EEG T3-Fz coherence and balance performance. We identify several reasons for this discrepancy. However, we conclude that, regardless of the underlying mechanisms, the current results indicate that we cannot assume that basic concepts associated with internal focus and motor performance (10) are easily transferrable to different cohorts/populations, especially those influenced by age-related changes.

## Author Contributions

VC and TW conceived this study. TE and WY designed the study. VC collected the data. VC, TE, and TM analyzed the data. All authors interpreted the results and wrote the manuscript. All authors read and approved the final manuscript.

### Conflict of Interest Statement

The authors declare that the research was conducted in the absence of any commercial or financial relationships that could be construed as a potential conflict of interest.

## Footnotes

^1^Participants also completed an additional two 20s trials under conditions of External focus, whereby they were instructed to keep the pole as stable and level as possible. However, as these instructions often resulted in participants visually fixating the pole (unlike the fixations made toward the distant wall during Baseline and Internal focus), we deemed that these between-condition differences in gaze distance would likely confound results. Consequently, data from the External focus condition have not been included in the analysis.
